# Evaluating GPT-4’s Cognitive Functions Through the Bloom Taxonomy: Insights and Clarifications

**DOI:** 10.2196/56997

**Published:** 2024-04-16

**Authors:** Kuan-Ju Huang

**Affiliations:** 1 Department of Obstetrics and Gynecology National Taiwan University Hospital Yunlin Branch Yunlin County Taiwan

**Keywords:** artificial intelligence, ChatGPT, Bloom taxonomy, AI, cognition

We are inspired by Herrmann-Werner et al’s [1] article, which assesses GPT-4’s cognitive functions based on the Bloom taxonomy. Adopting the Bloom taxonomy for evaluating GPT-4’s understanding of specific knowledge, traditionally applied to humans, is a novel concept. The results could also offer insights into whether GPT-4 can think like a human. However, some points in this article need clarification.

First, in Figure 3, the difficulty of the questions might have been inversely reported in the abstract, with 0 representing a very difficult question and 1 representing a very easy question, according to the description in the Quantitative Data Analysis subsection of the Methods. Consequently, GPT-4 performed better on easy questions than on hard ones.

Second, since a large language model (LLM) like GPT-4 operates by predicting the next word from its memory-based archive [2], it seems unlikely that GPT-4 would perform worst in the “remember” domain of the Bloom taxonomy in this study (42.65%) and excel in higher cognitive domains such as analyze, evaluate, and create, with incorrect reasoning accounting for 0%, 0.15%, and 0%, respectively, as reported in Table 3 [1]. The Bloom taxonomy categorizes the aims of questions, not the answers, in evaluating a “student’s” cognitive level within specific domains. Therefore, evaluating GPT-4’s cognitive functions by analyzing its responses presupposes that GPT-4 can think like a human. However, given our current understanding of how LLMs generate answers—essentially predicting the next word based on probabilities within a database—it is doubtful that GPT-4’s cognitive levels in responses can be accurately assessed using the Bloom taxonomy, especially with high scores in advanced cognitive domains [2].

For example, when evaluating “memory” (eg, definitions, guidelines, or facts), if the combination of elements exists in its database, GPT-4 can readily produce the most likely answers from its “memory.” Conversely, when elements are incorrectly combined, it may produce “hallucinated” answers [2]. In complex questions that test higher cognitive domains (eg, analyzing a previously unpublished case report with findings from subjective and objective medical evaluations to deduce the most likely diagnosis), if a similar case or key elements exist in GPT-4’s database, it might still produce a result from its “memory,” seemingly “analyzing, evaluating, and creating” an answer as it has “learned” from human problem-solving in similar cases. This “memory” function, considered LLM’s most potent capability compared to humans, can yield incorrect answers if the “memory” does not exist in the database (eg, news) or is not predicted as the next word. The apparent high cognitive function might result from the model’s ability to extract multiple human thought processes about a specific question from its vast database, akin to a well-trained system mimicking human cognitive processes [3,4].

Since most medical qualifying exams consist mainly of “memory” tests, the actual count of incorrect reasoning in the “memory” domain could be lower when both correct and incorrect answers are combined. Until more evidence proving that LLMs can think like humans is available, evaluating LLM-generated answers through the Bloom taxonomy may yield misleading results.

## Abbreviations

LLM: large language model
